# Ground reaction force metrics are not strongly correlated with tibial bone load when running across speeds and slopes: Implications for science, sport and wearable tech

**DOI:** 10.1371/journal.pone.0210000

**Published:** 2019-01-17

**Authors:** Emily S. Matijevich, Lauren M. Branscombe, Leon R. Scott, Karl E. Zelik

**Affiliations:** 1 Department of Mechanical Engineering, Vanderbilt University, Nashville, TN, United States of America; 2 Department of Orthopaedics, Vanderbilt University, Nashville, TN, United States of America; 3 Department of Biomedical Engineering, Vanderbilt University, Nashville, TN, United States of America; 4 Department of Physical Medicine & Rehabilitation, Vanderbilt University, Nashville, TN, United States of America; University of Colorado Boulder, UNITED STATES

## Abstract

**Introduction:**

Tibial stress fractures are a common overuse injury resulting from the accumulation of bone microdamage due to repeated loading. Researchers and wearable device developers have sought to understand or predict stress fracture risks, and other injury risks, by monitoring the ground reaction force (GRF, the force between the foot and ground), or GRF correlates (e.g., tibial shock) captured via wearable sensors. Increases in GRF metrics are typically assumed to reflect increases in loading on internal biological structures (e.g., bones). The purpose of this study was to evaluate this assumption for running by testing if increases in GRF metrics were strongly correlated with increases in tibial compression force over a range of speeds and slopes.

**Methods:**

Ten healthy individuals performed running trials while we collected GRFs and kinematics. We assessed if commonly-used vertical GRF metrics (impact peak, loading rate, active peak, impulse) were strongly correlated with tibial load metrics (peak force, impulse).

**Results:**

On average, increases in GRF metrics were not strongly correlated with increases in tibial load metrics. For instance, correlating GRF impact peak and loading rate with peak tibial load resulted in r = -0.29±0.37 and r = -0.20±0.35 (inter-subject mean and standard deviation), respectively. We observed high inter-subject variability in correlations, though most coefficients were negligible, weak or moderate. Seventy-six of the 80 subject-specific correlation coefficients computed indicated that higher GRF metrics were not strongly correlated with higher tibial forces.

**Conclusions:**

These results demonstrate that commonly-used GRF metrics can mislead our understanding of loading on internal structures, such as the tibia. Increases in GRF metrics should not be assumed to be an indicator of increases in tibial bone load or overuse injury risk during running. This has important implications for sports, wearable devices, and research on running-related injuries, affecting >50 scientific publications per year from 2015–2017.

## Introduction

Tibial stress fractures are a common type of overuse injury, associated with the accumulation of bone microdamage due to repeated submaximal loading that causes mechanical fatigue [1,2]. There is a high prevalence of tibial stress fractures in military recruits [3], recreational and elite runners [4,5], and other athletes [6–8]. Tibial stress fractures result in pain, healthcare costs and reduced physical activity [8,9]. Moreover, because recovery from tibial stress fracture typically requires rest and/or ankle immobilization (often for 6–12 weeks), this injury commonly results in missed work, decreased productivity, and physiological distress [10].

Factors that influence bone stress injury risk include the bone load intensity (magnitude, direction and duration of load), the rate of bone remodeling (influenced by length of activity and length of rest), and intrinsic factors (age, gender, bone density, geometry, mineral content, etc.) [11]. One potential way to reduce the incidence of bone stress injuries may be to monitor one or more of these risk factors in daily life, use bone fatigue models to estimate the damage accumulation, and then preemptively alert individuals of excessive damage accumulation. This approach might empower individuals, for instance runners, to modify training and allow the bone time to remodel and recover before an injury occurs. The challenge lies in how to implement this preventative solution, since direct measurements of bone load intensity, bone remodeling, and intrinsic factors are impractical in daily life. In the context of tibial stress fractures, monitoring load intensity might be realized via indirect estimates: using wearable sensors that are capable of estimating tibial bone force.

In the scientific literature, a number of lab-based motion analysis studies have sought to understand and predict overuse injury risks (to the tibia and other internal structures) by monitoring ground reaction force (GRF), as measured by a force plate under the foot [12,13]. Increases in GRF metrics are routinely assumed to reflect increases in internal structure loading (e.g., tibial bone loading). In an attempt to apply this approach outside of the laboratory, a growing number of consumer wearable devices–targeted largely towards runners and athletes–have been developed that use sensors capable of capturing features or correlates of the GRF. Commonly, wearable devices use one or more of the following: (i) pressure-measuring insoles, which capture localized forces acting normal to the surface of each sensor, and can be summed to estimate a component of the GRF, (ii) accelerometers mounted on the foot or shank, which can provide a correlate of GRF impact peaks [14,15] or loading rates [16], or (iii) accelerometers mounted on the pelvis, which can be analyzed to approximate the GRF active peak that occurs in midstance of running [17,18]. Commercial wearable devices then attempt to use these GRF-correlated signals to provide musculoskeletal loading or injury risk feedback to the user.

One limitation of current wearable devices (i.e., research and consumer wearables), as well as with the scientific running literature motivating them, is that they aim to understand, predict or prevent overuse injury risks solely by monitoring GRFs (or GRF correlates). However, GRF (the force between the shoe and the ground) is not the force experienced by structures inside the body, such as bones, muscles or joints [19–23]; and therefore GRF is not necessarily reflective of the actual repetitive loading that causes overuse injury to these internal structures. From a biomechanical perspective, there are several reasons why monitoring GRF to understand tibial bone loading or risk of tibial stress fracture is potentially problematic, a few of which are summarized below.

**Fig 1:**
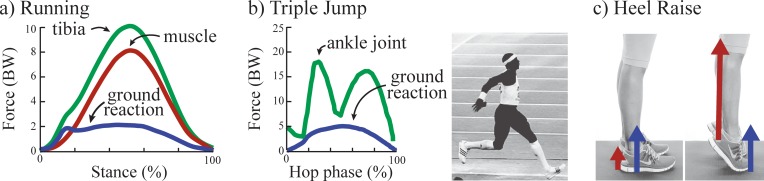
Examples of GRFs vs. tibial bone loading. (A) Tibial bone compression force (green) is much larger than GRF (blue) during running due to forces from muscle contractions (red); adapted from [21]. Forces are reported in body weights (BWs). (B) Peaks in tibial force (at the ankle joint, green) do not temporally coincide with peaks in GRF (blue) during the triple jump; adapted from [25]. Note, the GRF impact peak is not depicted here because it was not reported in this prior study, but it would have occurred at 0% of the cycle. (C) Standing flat footed vs. standing on one's toes results in the same GRF (blue), but different tibial forces, due to calf muscle contraction force (red) [26].

First, the load on the tibial bone is generally much larger than the GRF. This is because the vast majority of bone loading is due to muscle contractions during locomotion, not due to GRF; a fundamental insight derived from the work of Giovanni Borelli in the 17th century [19]. During running, peak GRFs are typically 2–3 times body weight, whereas peak forces on the distal end of the tibia are typically 6–14 times body weight, as evidenced by gait analysis ([21,23], **Fig 1A**) and modeling studies [20,24]. Likewise, a cadaver study that simulated walking using a robotic gait simulator found peak GRFs of 1.1 times body weight, measured with a force platform under the foot, and peak tibial compression force of 4.1 times body weight, measured with a force transducer directly in series with the tibia [22].

Second, peaks in GRF often do not coincide temporally with peaks in bone force. A GRF peak in running and jump landing often occurs at foot contact (impact peak), but tibial bone loading is typically small at this time. This is evidenced by *in vivo* bone stress and strain measurements [27], instrumented cadavers [22], musculoskeletal models [20], gait analysis studies [21,28] and data from instrumented joint implants [29]. Peak tibial load in running generally occurs later in the movement cycle, near midstance, and is closer in timing to (though not necessarily coincident with) the second peak of the GRF (often termed *active peak*). In the triple jump, two peaks in ankle joint (distal tibia) contact force have been estimated to happen at very different times than the impact and active peaks in the vertical GRF during the hop phase, due to muscle forces around the joint (**Fig 1B**, [25]).

Third, increases in tibial bone forces can occur without increases in GRF [30]. For example, standing flat-footed vs. standing on one’s toes results in the same GRF magnitude, but the latter can have much higher bone force due to calf muscle forces (**Fig 1C,** [26]). The GRF and tibial force are related through equations of motion [20,21] which depend on other time-varying factors such as the center-of-pressure under the foot, segment orientations, muscle contraction forces, and the direction of the GRF vector. There may be a subset of activities when increases in GRF metrics are indicative of increases in tibial bone loading; however, this is only expected in very special cases (e.g., if all the other terms in the equation of motion are constant, or nearly constant, or if changes in terms uniquely offset each other as to have negligible effect on total bone loading for a given subset of activities).

Despite these limitations, the use of GRF metrics (e.g., peaks, loading rates) or correlates from wearable sensors (e.g., tibial shock) remains popular amongst researchers and commercial device developers aimed at identifying and reducing overuse injury risks. The advantage of using GRF metrics is that they are easy to measure non-invasively in the lab using force plates, or outside the lab with portable wearable devices (which are relatively cheap and easy to integrate into shoes and clothing). However, a key question remains unanswered: is running a special case, such that increases in GRF are strongly correlated with increases in tibial bone load? If so, then GRF metrics (or GRF-correlates from pressure-insoles or accelerometers) may indeed serve as a useful tool for monitoring tibial bone loading changes during running, supporting the approaches currently used in scientific research and commercial wearable devices. If not, then it would dissuade the use of GRFs as a surrogate for tibial bone loading, and suggest the need to move beyond GRF measures (and GRF-correlates) alone in order to effectively monitor overuse injury risks in daily life. The purpose of this study was to determine if higher GRFs were indicative of (i.e., strongly correlated with) higher tibial bone loads when running over a range of speeds and ground slopes. Because of the complex relationship between GRF and internal bone loading, we hypothesized that increases in common GRF metrics (impact peak, loading rate, active peak, impulse) would not be strongly correlated with increases in tibial bone load metrics (peak force and impulse) across this range of running conditions (i.e., r<0.8).

## Methods

Ten healthy subjects participated who each reported that they run a minimum of 10 miles per week (5 male, 5 female; age: 24±2.5 years; height: 1.7±0.1 m; mass: 66.7±6.4 kg). All subjects gave written informed consent to the protocol, which was approved by the Institutional Review Board at Vanderbilt University.

**Fig 2:**
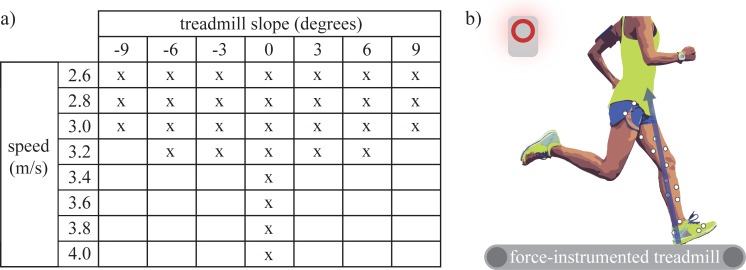
Summary of methods. (A) Each subject performed 30 running trials at a combination of speeds and slopes. (B) Experimental protocol involved subjects running on a force-instrumented treadmill while GRFs (blue vector) and lower-limb kinematics were recorded (white circles represent motion capture markers).

We selected a subset of running conditions that a recreational runner might encounter on a daily run. A fully comprehensive condition set (i.e., all plausible combinations of speed, ground slope, step frequency, footstrike pattern, footwear, terrain stiffness, fatigue level, etc.) was not feasible to test in lab. Thus, we had to select a subset of conditions to explore. We note that typical speeds and slopes will be different for each individual runner and across different runs/days (based on their fitness, training environment, etc.). Since there are no definitive criteria by which to select the subset of conditions, we chose a range of speeds and slopes that we felt was reasonable, practical and relevant based on the recreational runners we planned to test (**Fig 2A**). In an effort to maximize generalizability, each runner performed the same set of conditions. At slower speeds (2.6–3.0 m/s), we swept across the broadest range of slopes, from -9 to +9 degrees (**Fig 2A**). The highest speeds (3.4–4.0 m/s) were only performed on level ground to ensure all runners could complete the same conditions, to help limit the total number of conditions and to mitigate confounds due to fatigue (which we considered an interesting but separate investigation). Subjects wore their own personal running shoes. Each condition was performed on a treadmill for at least 30 seconds; ~10 seconds to adjust to the speed and slope, then data were recorded for 20 seconds. Breaks were taken between trials to adjust the treadmill slope, or if the subject requested a break for any reason.

We collected lower-limb kinematics and GRFs (**Fig 2B**). Kinematics were collected at 100 Hz (Vicon), then low pass filtered at 10 Hz (3rd order, zero-lag Butterworth). Four markers were placed on each segment (thigh, shank, foot), 2 on the lateral and medial femoral epicondyles, and 2 on the lateral and medial malleoli. Functional joint centers, segment angles, and joint moments were computed using C-Motion Visual3D software. The GRFs under each foot were collected at 1000 Hz using a force-instrumented treadmill (Bertec). The GRFs were low-pass filtered at 15 Hz (3rd order, zero-lag Butterworth) prior to computing inverse dynamics, similar to [31]. However, to avoid smoothing out GRF impact transients, these data were low-pass filtered at 45 Hz (3rd order, zero-lag Butterworth) for extracting GRF metrics, similar to [32]. For each trial, individual stance phases were parsed out, outcome metrics (as detailed below) were computed on a step-by-step basis, and then averaged.

**Fig 3:**
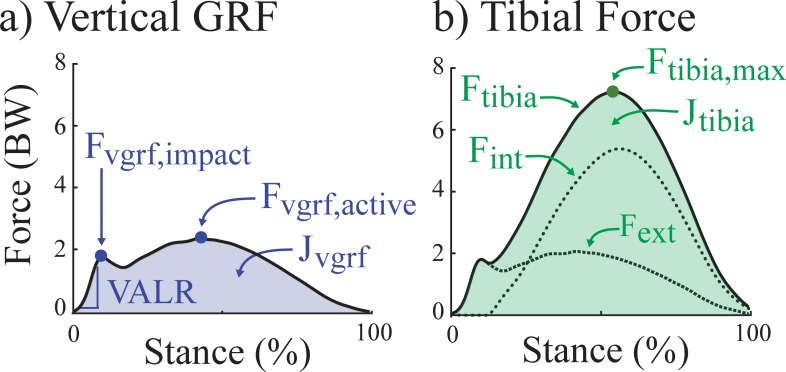
Outcome metrics. (A) Four commonly-used vertical GRF metrics: F_vgrf,impact_: impact peak; VALR: vertical average loading rate; F_vgrf,active_: active peak; J_vgrf_: total vertical impulse. (B) Two tibial bone force metrics: F_tibia,max_: maximum tibial compression force; J_tibia_: tibial compression force impulse. Two additional force estimates are shown for reference: F_ext_: the contribution of the external GRF to tibial compression; F_int_: the contribution of internal muscle force to tibial compression.

We computed four vertical GRF metrics that are commonly reported in the running literature, with vertical defined with respect to the absolute lab reference frame (i.e., parallel to the gravity vector): F_vgrf,active_ (vertical GRF active peak), F_vgrf,impact_ (vertical GRF impact peak), VALR (vertical GRF average loading rate) and J_vgrf_ (vertical GRF impulse) (**Fig 3A**). F_vgrf,active_ was defined as the maximum vertical GRF during 40–60% stance. F_vgrf,impact_ was defined as the local maximum peak of vertical GRF between foot contact and F_vgrf,active_. Foot contact was defined as when vertical GRF increased above 20 N. If an impact peak was absent in more than half of the gait cycles for a trial, then average F_vgrf,impact_ was not calculated for that running condition. The number of running conditions for which a subject did display an impact peak in more than half the gait cycles was also recorded. The VALR was estimated as the change in vertical GRF for the first 25 ms after reaching a threshold of 50 N, a method that does not rely on the presence of an impact peak [33,34]. J_vgrf_ was calculated as the time integral of the vertical GRF over stance.

Tibial bone load over the stance phase of gait was calculated as the longitudinally compressive force on the distal end of the tibia, a common location for stress fractures in runners [35]. The total force on the ankle (i.e., distal tibia) was calculated using a lower limb model, similar to prior studies (e.g., [21]): by summing the net force on the ankle (F_ext_) plus an estimate of force from the calf muscles generating torque about the ankle (F_int_). Ankle force was assumed to be indicative of tibial bone loading [36]. Net force on the ankle was estimated as the 3D GRF projected onto the long axis of the tibia, estimated as the vector connecting the ankle joint to the knee joint. In this calculation, foot mass and inertia were assumed to be negligible to avoid underestimating contributions from the GRF due to measurement errors in modeling or tracking foot segment motion; though net ankle force estimates using an anthropometric foot mass are very similar (typically within ~0.1 body weight based on an informal sensitivity analysis of our own data). Calf muscle force contribution was estimated as the sagittal plane ankle moment divided by the Achilles tendon moment arm, assumed constant (5 cm, [37,38]). We used this simplified model because it has been previously shown to yield Achilles force estimates during running that were similar to Achilles force estimates from a musculoskeletal model using 300 muscles with static optimization that minimized sum of muscle forces (e.g., peak tendon forces within ~4%, [39]). Studies that included other muscle groups when estimating tibial compression found a small tibial compressive force contribution from dorsiflexors (<0.5 body weight, [20,24]), but this force only existed for 0–20% and 90–100% stance, and a small compressive force contribution from other plantarflexors (<0.35 body weight, [20]). The lower limb model used has produced results that are qualitatively consistent with *in vivo* tibial strain measurements [27] and direct tibial bone load measurements in cadavers [22].

We then computed two tibial bone load summary metrics: F_tibia,max_ (maximum tibial compression force), J_tibia_ (tibial compression force impulse) (**Fig 3B**). F_tibia,max_ was defined as the peak tibial bone force over stance phase. J_tibia_ was calculated as the time integral of the tibial bone force over stance. These metrics were selected because of their relevance to the load intensity (magnitude and time duration of loading): maximum force magnitude is relevant to cyclic fatigue and force impulse is relevant to creep damage accumulation or cumulative load over time or distance [1,40–46].

The Pearson correlation coefficient (r) was computed for each GRF metric versus each tibial force metric on a subject-by-subject basis across all running conditions. The inter-subject range of correlation coefficients was identified and average correlation coefficients across subjects were computed using Fisher’s z transformation [47]. Force data were normalized by subject body weight for reporting purposes. A *strong positive* correlation was defined here as r≥0.8, *moderate positive* correlation as 0.5≤r<0.8, *weak positive* correlation as 0.3≤r<0.5, *negligible* correlation as -0.3<r<0.3, *weak negative* correlation as -0.5<r≤-0.3, *moderate negative* correlation as -0.8<r≤-0.5, and a *strong negative* correlation as r≤-0.8.

## Results

**Fig 4:**
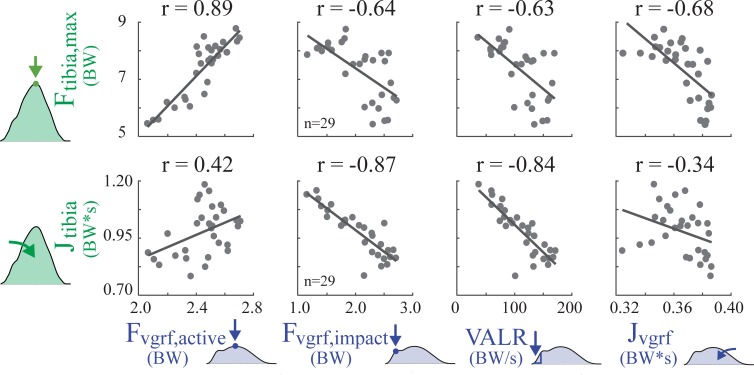
Regression results for GRF metrics vs. tibial bone load metrics across 30 running trials, for a single subject (Subject 1). Each gray dot represents a single condition (i.e., a given speed and slope from **Fig 2**), and *n* indicates number of running conditions that exhibited a measurable GRF impact peak for Subject 1. The correlation coefficient (r) was computed for Subject 1 across all running speeds and slopes. Note that no single subject should be considered representative given the large inter-subject variability observed. For instance, the one strong correlation shown for this subject (r = 0.89) was as low as r = 0.16 for another subject. The correlation coefficients for each individual subject are reported in **Table 1**.

On average, none of the GRF metrics were strongly correlated to tibial force metrics (**Table 1**, **Fig 4**); nor were there any GRF metrics for which the majority of subjects exhibited strong positive correlations with either of the two tibial load metrics.

**Table 1:** Correlation coefficients (r) between GRF metrics and tibial bone load metrics *across all trials within a subject*. Ten rows represent the 10 subjects (F = female, M = male). Within a subject, (n) indicates the number of running conditions (of 30 total conditions) that exhibited a measurable GRF impact peak (i.e., evident in more than half the gait cycles). Mean and standard deviation (std) were computed using Fisher’s z transformation.

Subject	F_vgrf,active_	F_vgrf,impact_	VALR	J_vgrf_	F_vgrf,active_	F_vgrf,impact_	VALR	J_vgrf_
	F_tibia,max_				J_tibia_			
1 (F)	0.89	-0.64, n = 29	-0.63	-0.68	0.42	-0.87, n = 29	-0.84	-0.34
2 (F)	0.84	-0.47, n = 27	0.23	-0.02	0.58	-0.78, n = 27	-0.10	0.12
3 (F)	0.72	-0.06, n = 10	0.01	-0.36	-0.17	-0.62, n = 10	-0.80	-0.17
4 (M)	0.90	0.27, n = 19	0.13	-0.20	0.60	-0.14, n = 19	-0.27	-0.20
5 (F)	0.72	-0.33, n = 24	-0.30	-0.66	0.13	-0.87, n = 24	-0.80	-0.48
6 (F)	0.58	-0.45, n = 19	0.13	-0.49	-0.60	0.30, n = 19	-0.91	0.07
7 (M)	0.85	-0.24, n = 22	-0.44	-0.68	0.47	-0.78, n = 22	-0.81	-0.53
8 (M)	0.26	0.34, n = 15	-0.24	-0.36	-0.54	-0.13, n = 15	-0.72	-0.07
9 (M)	0.16	-0.46, n = 16	-0.65	-0.84	-0.65	-0.10, n = 16	-0.80	-0.34
10 (M)	0.63	-0.64, n = 17	0.00	0.19	0.09	-0.14, n = 17	-0.62	0.74
mean±std	0.72 ± 0.42	-0.29 ± 0.37	-0.20 ± 0.35	-0.46 ± 0.40	0.03 ± 0.51	-0.51 ± 0.53	-0.72 ± 0.41	-0.11 ± 0.41
[min max]	[0.16 0.90]	[-0.64 0.34]	[-0.65 0.23]	[-0.84 0.19]	[-0.65 0.60]	[-0.87 0.30]	[-0.91–0.10]	[-0.53 0.74]

### Active peak

F_vgrf,active_ was positively or negligibly correlated with F_tibia,max_ in all subjects (0.72 ± 0.42): two exhibited negligible correlations, four individuals exhibited moderate correlations, and four exhibited strong correlations. F_vgrf,active_ had an inconsistent relationship with J_tibia_ (0.03 ± 0.51): four subjects showed a moderate positive correlation, three showed a moderate negative correlation, and three showed negligible correlation.

### Impact peak

F_vgrf,impact_ was on average negatively correlated with F_tibia,max_ (-0.29 ± 0.37): three subjects exhibited a negligible correlation, four exhibited a weak negative correlation, one exhibited a weak positive correlation, and two exhibited moderate negative correlations. F_vgrf,impact_ was on average negatively correlated with J_tibia_ (-0.51 ± 0.53): four subjects showed a negligible correlation, one showed a positive moderate correlation, three showed a moderate negative correlation, and two showed a strong negative correlation. On average, measurable GRF impact peaks were only observed for 20 ± 6 conditions for each subject. The majority of subjects were rearfoot strikers and had measurable GRF impact peaks during level and decline running; however, most individuals changed their footstrike pattern on more inclined slopes and the impact peaks tended to disappear. Across all subjects, measurable GRF impact peaks were present during 71 of 80 level running trials and during 98 of 110 decline trials, but only during 29 of 110 incline trials.

### Loading rate

The correlation between VALR and F_tibia,max_ was generally weak or negligible, but varied considerably between subjects (-0.20 ± 0.35): six subjects exhibited a negligible correlation, two exhibited a weak negative correlation, and two exhibited a moderate negative correlation. The VALR and J_tibia_ were negatively or negligibly correlated in all subjects (-0.72 ± 0.41): two subjects showed a negligible correlation, two showed a moderate negative correlation, and six showed a strong negative correlation.

### Impulse

The correlation between J_vgrf_ and F_tibia,max_ varied across subjects (-0.46 ± 0.40): three subjects exhibited a negligible correlation, two a weak negative correlation, four a moderate negative correlation, and one a strong negative correlation. The correlation between J_vgrf_ and J_tibia_ also varied across subjects (-0.11 ± 0.41): five subjects showed a negligible correlation, four showed a moderate negative correlation and one showed a moderate positive correlation.

### Discussion

We found that increases in GRF metrics were not strongly correlated with increases in tibial bone loading metrics during running across speeds and slopes (**Table 1**); rather most correlations were negligible, weak, or moderate. Although there was high inter-subject variability in the strength of correlation, 76 of the 80 subject-specific correlation coefficients supported our hypothesis. The only strong positive correlations were in 4 of 10 subjects between F_tibia,max_ and F_vgrf,active_. Also of note, 2 subjects showed a strong *negative* correlation between J_tibia_ and F_vgrf,impact_, and 6 showed a strong *negative* correlation between J_tibia_ and VALR.

**Fig 5:**
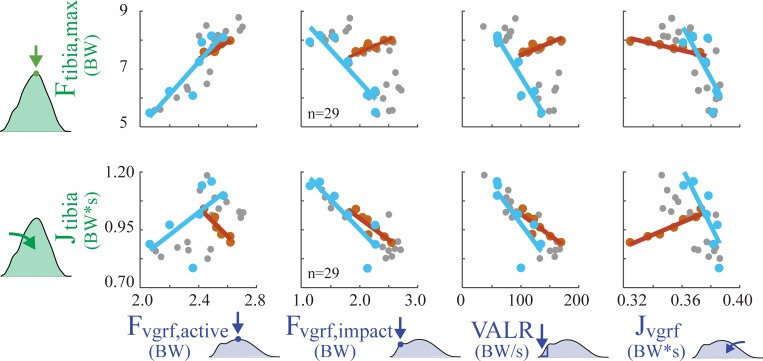
Force trends due to changing speed vs. changing slope for a single subject (Subject 1). Lines represent regression results for GRF metrics vs. tibial bone load metrics when only speed or slope was varied. Dark orange dots represent conditions when speed was varied while running on a fixed slope (level ground). Light blue dots represent conditions when ground slope was varied while speed is held constant (at 2.6 m/s). Small gray dots are all remaining parameter sweep conditions.

Isolating effects due to speed and slope provides some insights into why GRF metrics were not strongly correlated with tibial loading (**Fig 5**). When runners encountered a change in ground slope, relationships between GRF and tibial loading often changed drastically. For instance, F_vgrf,impact_ and VALR were often positively correlated as speed increased on level ground, but typically switched to having a negative correlation across a range of slopes when speed was held constant (**Fig 5**). F_vgrf,impact_ and VALR metrics both decreased with increasing ground slope (similar to [48]), however F_tibia,max_ increased due to higher muscle forces. Likewise, J_vgrf_ and J_tibia_ were positively correlated as speed increased on level ground, but were negatively correlated when slope changed at a single fixed speed.

These findings highlight that there are only limited special cases when GRF metrics are strong indicators of tibial bone load. When ground slope was held constant at zero degrees (level), then as speed increased all subjects showed a strong positive correlation between F_vgrf,active_ and F_tibia,max_ (r = 0.97, inter-subject mean computed in post-hoc analysis). Further, F_tibia,max_ was also moderately to strongly correlated to F_vgrf,impact_ (r = 0.90) and VALR (r = 0.90) in all subjects during running on the zero-degree slope. However, even at this fixed slope, some runners exhibited weak relationships between GRF metrics and J_tibia_. When looking at level and decline running conditions together, the correlation between F_tibia,max_ and F_vgrf,impact_ (r = -0.19) and between F_tibia,max_ and VALR (r = 0.45) became negligible and weak; though F_vgrf,active_ and F_tibia,max_ remained strongly correlated (r = 0.94). Once level, decline, and incline conditions were all analyzed together the correlation between F_vgrf,active_ and F_tibia,max_ became moderate (r = 0.72). Note that the special cases when GRF metrics were strongly correlated to tibial bone load are not necessarily well-suited for real-world outdoor running, in which a runner who encounters a decline generally also encounters an incline if they aim to start and end at the same location.

From this very simple set of running conditions (i.e., varying only speed and slope), it is evident why increases in GRF metrics generally should not be assumed to be a surrogate for, or indicator of, increases in tibial bone loading. During real-world training, additional confounds such as changes in muscle coordination or running pattern (e.g., due to fatigue, soreness, terrain, shoe properties, footstrike pattern, etc.), may further alter the relationship between GRF and tibial bone load.

### Key implications and discussion related to scientific research

The lack of strong correlations in this study suggest that GRFs provide limited insight into tibial bone loading during running across speeds and slopes, and therefore may provide limited utility for understanding or predicting overuse injury risk associated with this repetitive internal structure loading. Running GRFs have been analyzed in many ways in the scientific literature over the last several decades –extracting impact peaks, loading rates, active peaks, and impulses from vertical GRF, analyzing peaks from fore-aft GRFs, quantifying GRF frequency content, etc.–in the hopes that some feature of this force between the foot and ground might indicate injury risk, such as tibial stress fracture risk. However, GRFs provide an incomplete perspective on musculoskeletal loading and may be the wrong signal to be monitoring/analyzing if we seek to understand tibial bone load or assess overuse injury risk. This is of broad concern to the research field given the substantial time and resources invested into gait analysis and epidemiological studies that seek to relate bone stress injury to GRF metrics (or GRF correlates, e.g., based on accelerometers or pressure insoles), and given the pervasive influence these GRF metrics have had on sport science (e.g., comparisons of footwear or running patterns), training, coaching and product development (e.g., running shoes).

Results from this study reinforce previous experimental evidence and theoretical arguments against using or interpreting GRF impacts and loading rates to identify overuse injury risks [28,49–52]. Although some epidemiological studies have observed an association between high impacts or loading rates and running-related injuries [33,53–56], there are many studies that have failed to find such a correlation [30,57–62]. These conflicting results also exist between prospective studies (e.g., [53,54,61–64]). Several textbooks, review articles and commentaries further highlight this conflicting evidence [12,13,28,52,65,66]. Nevertheless, the use of higher GRF impact peaks and loading rates to infer injury risk remains extremely common in the scientific literature. Based on a literature search of articles published between 2015–2017, we discovered that during this period more than 50 peer-reviewed publications per year assume, report or interpret GRF impact peaks or loading rates to signify increased injury risk.

The reason for the sustained popularity of GRF metrics is likely multifaceted, but may stem partly from measurement convenience, or from people’s intuition based on how we as humans perceive external (vs. internal) loads on the body (though such intuitions can often be misleading in biomechanics, e.g., [19]), or simply from the GRF impact paradigm being deeply embedded in the running literature over recent decades. Another contributing factor may be related to misinterpretation and misapplication of prior bone mechanics studies [67,68]. Studies on rabbits and guinea pigs have shown that repeated impulsive loading can cause bone microdamage [69,70]. However, this finding has been applied in running-related injury studies to support a subtly but significantly different contention: that higher impact peaks (impulsive loading) are associated with higher injury risk (e.g., due to more microdamage accumulation) [32,71,72]. The critical thing to highlight is that this contention is only valid if we also assume that during running the impact loading is *the primary cause* of bone microdamage. To our knowledge, there is no evidence to support this assumption. During running, the majority of the tibial force is due to muscle contraction (**Fig 1A**). Note that the commonly-cited impulsive loading studies [69,70] did not compare damage due to impact forces relative to damage due to muscle forces, and therefore provide no experimental evidence that GRF impacts are the primary cause of bone microdamage. The relationship between the magnitude of force on a biological structure and the microdamage caused by the force is non-linear [1,73]. According to Miner’s rule, microdamage is roughly proportional to force to the *C* exponent (i.e., force ^*C*^) [11], where *C* is a bone-specific constant found experimentally via mechanical fatigue studies. We can apply this well-established relationship to estimate the relative amount of damage caused by forces at different parts of the gait cycle (e.g., impact vs. midstance). As depicted in **Fig 1**, the GRF impact peak is ~2 body weights, whereas the peak tibial force in midstance is ~8 body weights (which includes muscle contraction forces). Using the empirically-derived exponent for cortical bone of *C* = 7 [40,43,51], we estimate that on every step the peak tibial force at midstance would be expected to cause about sixteen thousand (8^7^/2^7^ ≈ 16,000) times more microdamage to bone than the GRF impact peak. Thus, the relative damage due to the impact forces may be trivially small regardless of whether impacts are slightly larger or smaller (e.g., 1.8 vs. 1.55 body weights, as reported in [74]). This evidence contradicts the prevailing belief that impacts are the source of overuse injuries, and further highlights why it generally should not be assumed that increases in GRF impact peak are reflective of increased injury risk.

A similar misinterpretation issue may underlie the use of loading rate (and strain rate) findings from bone mechanics studies. One commonly-cited study by Schaffler et al. [75] concluded that “cyclic loading at a higher physiological strain rate causes more damage than cyclic loading at a lower strain rate.” However, upon careful inspection of the results we discovered that this conclusion was not substantiated by the statistical analysis performed. The study compared the effects of high strain rate vs. an unloaded control, and the effects of low strain rate vs. an unloaded control; however, it did not directly compare the high vs. low strain rate groups. When we performed statistical analyses using the study results presented in the paper we failed to find significant differences between high vs. low strain rate groups for any of the reported outcome metrics; namely, bone stiffness loss (p = 0.07), number of microcracks (p = 0.32), density of microcracks (p = 0.28) and length of microcracks (p = 0.48). We compared bone stiffness loss using the Mann Whitney U test (the primary statistical test employed in the original study) since a table of specimen-specific results were provided. For the remaining metrics we performed a two-sample t-test using the mean ± standard error results reported in the publication (since specimen-specific results were not provided to perform a Mann Whitney U test). Inspection of the estimated 95% confidence interval for the difference in means of each outcome metric further substantiated our take-away that from these published data one cannot conclude that the higher loading rate caused more damage than the lower loading rate. Significance level of 0.05 was used for our interpretation, consistent with the threshold set in the original publication. Meanwhile, a more recent cyclic loading study on bone specimens found that high loading rates associated with GRF impacts in running had little effect on bone fatigue [51]. Likewise, bone samples loaded at higher rates have been observed to take more cycles to failure, suggesting less damage accumulation per cycle [1,40,46]. Collectively, this evidence seems to call into question the common assumption that higher loading rates indicate higher bone damage accumulation (or injury risk).

In summary, there are substantive concerns about how impulsive bone loading studies are commonly interpreted [67,68], and how this may misguide the use of GRF metrics like impact peak and loading rate. The field would benefit from a clear and careful synthesis of bone mechanics studies, to ensure this knowledgebase is being appropriately interpreted and applied in the assessment of overuse injury risks.

### Key implications and discussion related to wearable devices

While motion analysis and musculoskeletal modeling methods have allowed researchers in the lab to estimate forces on certain internal structures, recreating these estimates outside the lab with non-invasive, low-cost, and portable sensors remains a grand challenge in the biomechanics field. Most commercial devices use GRF-correlated metrics (e.g., tibial shock) from accelerometers and/or pressure insoles to provide loading or injury risk feedback to the user. A key underlying assumption of these devices is that increases in GRF metrics reflect increases in loading inside the body. For instance: (i) IMeasureU outputs a “bone load” metric that increases with “the size of the [ground] impact derived from each individual step,” based on the stated rationale that impact peaks “can function as a surrogate measure of the loads experienced by the underlying musculoskeletal tissue” [76,77], (ii) Runscribe outputs “Impact Gs” and indicates that lower “Impact Gs” at footstrike may help prevent injuries [78], (iii) MileStone states that a “low rate of impact… is optimal and can help prevent injury” [79], (iv) Stridalyzer states that “‘Pounding’ [the foot] during landing… can increase impact forces, which over time leads to injuries” [80], and (v) Sensoria provides an "Impact Score,” stating it is “a quantitative relative measure, on a scale from 1 to 10, driven by the impact forces generated when your foot hits the ground while your run. In order to reduce likelihood of injury, you want to keep your impact score as low as possible” [81]. However, the key assumption underlying each of these statements/claims remains unsubstantiated for running, both experimentally and from a theoretical standpoint. Tibial acceleration may be correlated with tibial load around impact (i.e., over the first ~40 ms after foot-ground contact [82]) when forces are relatively low (**Fig 1A**). However, as shown in this study, commonly-use GRF metrics (e.g., impact peak, loading rate) are not necessarily reflective of the much larger tibial bone forces experienced later in the gait cycle, nor is there a biomechanical rationale or consistent epidemiological evidence to support interpretations of these GRF metrics or correlates like tibial shock as indicators of injury risk.

Presently there is a lack of transparency and validation amongst consumer wearables [83]. Many commercial devices selectively cite studies that support their chosen outcome metric, while omitting published counter evidence. Others simply fail to provide scientific evidence that their outcomes are indicative of increased loading or injury risk to specific internal structures. As a result, biofeedback from existing wearable devices may be misleading users. Wearable devices often employ ambiguous terminology such as “limb load”, “step intensity” or “biomechanical load”–terms which do not specify which individual structure in the body, if any, experience the purported load. Some devices employ misleading terminology such as “bone load,” which has been defined as a weighted sum of impact peaks [76]. As seen in this study, footstrike impact peaks are not the main source of bone loading, and cannot be used as a surrogate to infer the peak force or force impulse experienced by the tibia. Some commonly-used metrics–like impact peaks and loading rates–may even be negatively correlated with bone loading during running (**Fig 4, Table 1**). This means the current interpretation of these values in wearable devices may be leading to the wrong conclusions about the accumulation of microdamage to a bone such as the tibia. Further ambiguity is introduced when commercial device metrics refer or allude to overall injury risk (i.e., to structures throughout the body). The idea of having a single or small number of output metrics that can capture overall injury risk is appealing to consumers, clinicians, researchers and wearable device manufacturers alike. However, there is no guarantee, nor theoretical basis, that this global injury metric is embedded within the GRF waveform (i.e., hidden within this relatively small force magnitude between the foot and ground).

The wearable device field would benefit from more deliberate and targeted attempts to monitor loading on specific internal structures at high risk of injury, with less emphasis on GRF metrics. There have been a number of innovative advances in sensing that provide estimates of loading on a given muscle or internal structure (e.g., [84]). These more targeted approaches offer the opportunity to better understand structure-specific loading, such that we can more confidently associate forces on a given structure (bone, muscle, tendon, etc.) with overuse injuries that may eventually develop in said structure. Moreover, estimating the time-varying force experienced by specific structures (as opposed to only computing discrete summary metrics related to peaks or impulses) may offer a more promising avenue of identifying and understanding specific injury risks in running and other activities. Given the complexity of human movement, and difficulty of measuring internal forces non-invasively, data from multiple wearable sensors may need to be fused in order to monitor load on certain internal structures *in situ*.

### Potential utility of GRF metrics

There may be situations when GRFs still provide some utility for understanding bone loading or overuse injuries. For instance, a given GRF metric might be useful in situations when the metric has been validated to be a strong indicator of loading on a specific internal structure (e.g., tibial bone) for a given individual (or subset of individuals) and for a given subset of activities (e.g., running over a specified range of speeds, slopes, etc.). However, at present, the majority of published studies seem to use and interpret GRF metrics without adequate subject- or activity-specific validation.

One potential use of GRF metrics might be for lab-based experiments performed only on level ground (or potentially a flat track, though we did not assess curvilinear running in this study), given that we found strong correlations with F_tibia,max_ for all 10 subjects (as detailed earlier in Discussion). However, in post-processing of our data we found that running speed itself was also strongly correlated with F_tibia,max_ on level ground (r = 0.97, similar to the correlation between F_vgrf,active_ and F_tibia,max_). In many cases it may be preferable and easier to monitor speed than GRF.

A second potential use of GRF metrics might be for an individual runner who is studied extensively to establish that over a specified set of running conditions (speeds, slopes, terrains, levels of fatigue, etc.), a given GRF metric is a good indicator of load on a specific bone. For example, two subjects in our study (Subjects 1 and 7) showed a strong positive correlation between F_vgrf,active_ and F_tibia,max_ and a strong *negative* correlation between VALR and J_tibia_ (**Table 1**). For these two subjects, increases in F_vgrf,active_ strongly indicated *increases* in F_tibia,max_, and increases in VALR strongly indicated *decreases* in J_tibia_ over the range of speeds and slopes tested. These two subjects demonstrate a scenario when specific GRF metrics might be utilized to indicate bone load, but only when (i) the relationship was validated for that subject and that subset of conditions, and (ii) the relationship was identified as being strongly positive or strongly negative for a given GRF metric. Subject-specific validation may be feasible for elite runners, but would likely be impractical or prohibitively expensive for many recreational runners due to the amount of instrumented gait analysis required to perform this validation.

A third potential use of GRF metrics would be for testing hypotheses in which there is an indirect relationship between GRFs and loading on specific internal structures. Here we summarize one example: Studies suggest that larger GRF impacts cause more energy to be dissipated through wobbling of muscles in the legs [85]. To maintain a given running speed, when energy is dissipated then it must be offset by positive work performed through active muscle contraction. This additional muscle work could be achieved by either higher peak muscle forces and/or applying muscle forces over longer periods of time. It therefore might be hypothesized that increases in GRF impacts lead to more energy dissipation, which then leads to increased loading magnitude or duration of certain muscles or bones. Note that in this example the GRF impact peak is not the source of high internal structure loading, as commonly assumed. Rather, large GRF impacts might conceivably help explain a mechanism by which higher internal structure loading could result (potentially at a different time in the stride cycle). As such, GRF metrics could be useful in situations when they are a core part of a testable hypothesis.

One additional consideration worth noting is that impact peaks were not present in about one third of the running conditions in our study (mostly on inclines), which may limit the utility of this metric in comparing across a broad range of conditions. Relatedly, estimating GRF loading rate from running strides with vs. without impact peaks may be capturing slightly different aspects of the gait dynamics [86]. This may also limit practical applications and interpretations of this metric across different running conditions, particularly those in which footstrike pattern changes. Finally, we remind that this discussion is in relation to overuse injury risk, specifically tibial stress fracture risk. There are of course many other situations and applications when GRFs are extremely useful (e.g., computing inverse dynamics), and more broadly, GRFs remain one of the most important measurements in the field of biomechanics.

### Limitations

The scope of this study was limited to estimating changes in bone load within each subject. As discussed in the introduction, additional factors also affect stress fracture risk. To assess injury risk between subjects, additional subject-specific information on bone remodeling and intrinsic factors (age, gender, bone density, geometry, nutrition, mineral content, etc.) may also be necessary. A limitation of this study (and nearly all gait analysis studies) is that we are unable to directly measure tibial bone loading. However, there is strong converging evidence from cadaver [22], implanted sensor [23,27] and musculoskeletal modeling studies [20,24] that the non-invasive estimates used provide a reasonable approximation of tibial bone loading. In particular, we have confidence in the trends predicted with changing speed and slope, and would not expect imperfect bone load estimates to alter any of the major conclusions or interpretations. Another limitation is that we only performed linear, univariate regression analysis because this appears to be the most common way that prior scientific studies and current wearable devices are using GRF metrics (or correlates) to infer musculoskeletal loads or injury risks. Another limitation is that we only quantified tibial compression load. In future studies, it would also be interesting to estimate bending, shear or torsional loads [20,21,87] which contribute to the stress/strain of the tibia, or to use advanced modeling techniques to estimate local stress/strain concentrations [1,42,87,88]. These loading patterns are all highly influenced by muscle forces; therefore, GRF metrics should not be assumed to reflect these loads either, unless validated for a given subset of activities. Because there are currently no wearable devices that can track tibial bone load (or localized stress) longitudinally in daily life it is not yet known (e.g., from prospective studies) which of these bone loading directions or metrics might be most useful. Given the large magnitude of the tibial compression load this seems like a reasonable candidate to explore, and more informative of bone loading than GRFs. A final limitation is that in order to make this study and analysis tractable we focused on a single bone and a single overuse injury. The specific conclusions drawn are all related to whether changes in GRF metrics are reflective of changes in loading on this particular bone. However, stress fracture and overuse injuries commonly occur in several other internal structures in the lower limb as well (e.g., calcaneus bone, Achilles tendon). The broader implication of our study is that loading on these other bones, muscles and tendons in the body—perhaps even loading on the vast majority of structures in the body—may be poorly understood by monitoring changes in GRF or GRF correlates alone.

### Conclusion

In summary, increases in GRF metrics should not be assumed to correlate with increases in bone loading, nor assumed to signify increased risk for tibial stress fractures. The high inter-subject variability in correlations further strengthens this general conclusion. For any individual, both subject- and task-specific validation would be needed to assess if GRFs provide useful insight on loading of the tibial bone, or other internal structures. This study has important implications for scientific research on running-related injuries, and for the development and validation of current and future wearable devices. Specifically, these findings demonstrate that the way GRF metrics are commonly interpreted as indicators of musculoskeletal loading and injury risk in literature is often flawed, and the application of these GRF metrics in scientific research, sports and wearable devices can be highly misleading. Although GRF metrics may be convenient to measure and may seem to intuitively "make sense" as a way to monitor loading on the musculoskeletal system, the results here and in prior literature reveal that commonly-used GRF metrics may provide very limited insight on, and may even mislead our understanding of, internal structure loading such as to the tibial bone. The GRF may simply be the wrong signal to be monitoring/analyzing if we seek to understand or predict overuse injury risk to the tibia, or to other bones, joints, muscles and tendons in the body. Summarized poetically:

You go for a run down the street.

You feel the ground force on your feet.

You may think these reveal

The bone loads that you’ll feel,

But this thinking is just incomplete.

The force due to ground reaction

May be a stress fracture distraction.

Don’t assume force on shoe

To mean tibia load too

Since bone load’s mostly from muscle contraction.
